# Estimated HIV Trends and Program Effects in Botswana

**DOI:** 10.1371/journal.pone.0003729

**Published:** 2008-11-14

**Authors:** John Stover, Boga Fidzani, Batho Chris Molomo, Themba Moeti, Godfrey Musuka

**Affiliations:** 1 Futures Institute, Glastonbury, Connecticut, United States of America; 2 National AIDS Coordinating Agency, Gaborone, Botswana; 3 African Comprehensive HIV/AIDS Partnership, Gaborone, Botswana; University of California San Francisco, United States of America

## Abstract

**Background:**

This study uses surveillance, survey and program data to estimate past trends and current levels of HIV in Botswana and the effects of treatment and prevention programs.

**Methods/Principal Findings:**

Data from sentinel surveillance at antenatal clinics and a national population survey were used to estimate the trend of adult HIV prevalence from 1980 to 2007. Using the prevalence trend we estimated the number of new adult infections, the transmission from mothers to children, the need for treatment and the effects of antiretroviral therapy (ART) and adult and child deaths. Prevalence has declined slowly in urban areas since 2000 and has remained stable in rural areas. National prevalence is estimated at 26% (25–27%) in 2007. About 330,000 (318,000–335,000) people are infected with HIV including 20,000 children. The number of new adult infections has been stable for several years at about 20,000 annually (12,000–26,000). The number of new child infections has declined from 4600 in 1999 to about 890 (810–980) today due to nearly complete coverage of an effective program to prevent mother-to-child transmission (PMTCT). The annual number of adult deaths has declined from a peak of over 15,500 in 2003 to under 7400 (5000–11,000) today due to coverage of ART that reaches over 80% in need. The need for ART will increase by 60% by 2016.

**Conclusions:**

Botswana's PMTCT and treatment programs have achieved significant results in preventing new child infections and deaths among adults and children. The number of new adult infections continues at a high level. More effective prevention efforts are urgently needed.

## Introduction

Botswana has one of the highest levels of HIV in the world. UNAIDS estimated that adult prevalence was about 24% in 2005, higher than any other country except Swaziland [1]. The epidemic has imposed a terrible burden due to lives lost, reduced quality of life and a large number of orphans. Since 2003 Botswana and its development partners have launched strong efforts to prevent the transmission of HIV from mothers to their children and to provide advanced treatment to those who need it. Information on epidemic trends is available from sentinel surveillance conducted annually at antenatal clinics around the country. Program statistics describe the expansion of the PMTCT and treatment programs [2].

Comprehensive assessments of the demographic impact of the epidemic were conducted for Botswana in 2000 [3] and 2006 [4]. This report expands and updates those analyses using the latest surveillance and program data as well as updated models developed by UNAIDS. It describes the use of these data to estimate national prevalence in Botswana and to assess the implications of that estimate for other indicators of interest, such as the number of people infected, the annual number of new infections, the number of people in need of ART, and the impact of the PMTCT and treatment programs.

## Methods

The HIV surveillance program conducts annual HIV surveys among women aged 15–49 years attending ante-natal clinics. Surveillance has been conducted annually since 1992 and now includes 24 sites [5]. Since these surveys measure HIV prevalence in pregnant women they do not represent prevalence in all adults, including both men and women. However, since ANC surveillance is conducted annually it does provide information on trends in prevalence that should be representative of those in the general adult population.

The Botswana AIDS Impact Survey II (BAIS II) conducted in 2004 measured the prevalence of HIV infection in the population aged 18 months and above [6]. Although only 61% of those interviewed in this survey agreed to provide a blood sample for HIV testing, the results of this survey are thought to provide a reasonably accurate measure of HIV prevalence in 2004.

The UNAIDS Reference Group on Estimates, Models and Projections has developed several tools to estimate national prevalence. One of these tools, the Estimation and Projection Package (EPP), is used in most countries in sub-Saharan Africa to estimate prevalence trends from surveillance and survey data in countries with generalized epidemics [7]. EPP works by fitting a simple epidemic model to surveillance data from multiple sites over time. Separate estimates are made for urban and rural prevalence and then combined to produce a national estimate.

The epidemic model uses four parameters to determine the prevalence trend over time: the start year of the epidemic, the initial force of infection, the proportion of the population at risk of infection and the rate of replenishment of the population at risk when it is depleted by AIDS deaths. EPP generates 50,000 to 200,000 epidemic curves by randomly selecting values of these four parameters from plausible distributions. Each of these curves is tested to see how well it fits the surveillance and survey data. A sample of curves is drawn from the full set with the likelihood of selection proportional to the goodness of fit. The result is a most likely curve that provides a point estimate of prevalence in each year and a range around the point estimate.

The estimates of adult HIV prevalence are used in the AIDS module of Spectrum [8] to estimate the other indicators of interest such as the number of people living with HIV, new infections, AIDS deaths, need for treatment and the number of orphans. HIV prevalence among adults 15–49 is combined with information on the age and sex distribution of prevalence from the national survey to estimate the distribution of prevalent adult infections by age and sex. The trend in prevalence is combined with information on the distribution of time from infection to AIDS death from cohort studies to estimate the number of new adult infections by age and sex. New infant infections are estimated from prevalence among pregnant women and the rate of mother-to-child transmission, which is dependent on infant feeding practices and the coverage of prophylaxis with ARVs. New infections progress over time to a symptomatic stage where ART is needed.

The rate of progression from infection to AIDS death for adults is a distribution drawn from cohort studies in sub-Saharan Africa [9]. The median time from infection to AIDS death without treatment is 11 years. Eligibility for ART is assumed to occur at a median of three years before AIDS death [10]. Those who receive first- and/or second-line ART experience extended survival. In Botswana, program data are available on the proportion of ART patients who are known to be alive by time since the initiation of therapy. As of December 2007 information is available on 75,393 patients. These data show that after 12 months, 91.3% of ART patients are known to be still alive, and after five year 86% of ART patients are still alive. These rates mayoverstate the true survival since some of those who were lost to follow-up (i.e. their status is unknown) have probably died. The UNAIDS Reference Group on Estimates, Models and Projections assumes that for most new programs the annual survival rates are 85% for the first year on ART and 95% for subsequent years [10]. However, the first year survival is likely to be lower in new programs with low ART coverage since most patients get started on ART very late, with low CD4 counts. When coverage is high as in Botswana patients are identified as being in need earlier and generally start ART at higher CD4 counts which should result in better first year survival.

People at any stage are subject to non-AIDS mortality at the same rates as those who are not infected. The progression of children from infection to AIDS death is modeled as a double Weibull curve fitted to longitudinal data [11]. AIDS and non-AIDS orphans are estimated from the number of adult deaths each year, the fertility history of those who die and the rates of child survival.

Spectrum estimates the uncertainty around the estimate of each indicator by using a Monte Carlo approach. One thousand projections are calculated using different prevalence curves provided by EPP and drawing random values of other key parameters (such as the progression time to AIDS death, and the effectiveness of ART) for each projection. The results are analyzed to produce 5% and 95% plausibility bounds around each point estimate. (These are not true confidence intervals since the errors around the input assumptions are not measured directly and we cannot include all sources of error.)

## Results

Results for key HIV/AIDS indicators are shown in Table 1. The plausibility bounds for prevalence and number of adults infected are narrow because the national survey provided an accurate estimate of the level of infection in 2004. Bounds for other indicators rely on additional assumptions, such as the progression period from infection to death and the effects of ART and PMTCT and, therefore, are wider.

**Table 1:** Key HIV/AIDS Indicators and Plausibility Bounds, 2007

Indicator	Value in 2007	Plausibility Bounds
HIV Adults+Children	330,000	318000–345000
HIV Adults 15+	311,000	299000–325000
HIV population-Children	19,561	18000–21000
Prevalence Adult	25.7	24.8–26.7
New HIV infections-Adult	18,000	12000–26000
New HIV Infections-Children	890	810–980
Annual AIDS deaths-Adult	7,400	5000–11000
Annual AIDS deaths-Children	790	600–1020
Need for ART-Adult (15+)	120,000	101000–136000
Need for ART-Children	7,400	6800–7800
Mothers needing PMTCT	15,000	13000–16000
AIDS orphans	90,000	76000–103000

### Prevalent Adult HIV Infections

**Figure 1:**
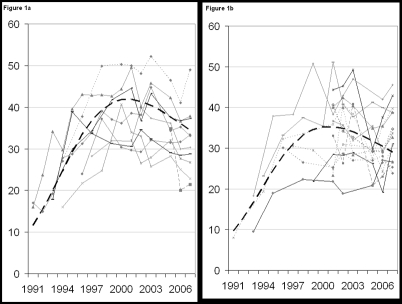
HIV prevalence among women attending antenatal clinics, 1991–2007. Data from urban clinics (Figure 1a) and rural clinics (Figure 1b) are shown in the gray lines. The smooth curve produced by EPP is shown in the dark dashed line for each region.

HIV trends at antenatal surveillance sites are shown for urban and rural sites in Figure 1. The best fitting curves to these data suggest that HIV prevalence has declined somewhat in urban areas since 2000 and has remained stable in rural areas. These curves need to be adjusted to match the national population survey of 2004. The urban curve needs to be adjusted downwards by 40% to account for the bias in urban surveillance sites compared to the entire urban population. The rural curve needs to be adjusted downwards by only 15%. In making this adjustment we assume that the ANC data represent the trend in prevalence and the national survey best represents the level.

When these curves are adjusted to match the national population survey of 2004 and weighted by the adult urban and rural populations they suggest that national prevalence among all adults 15–49 in Botswana peaked at about 26% in 2001 and has since declined slightly to 25.7% (24.8%–26.7%) by 2007. This implies that 280,000 adults 15–49 are currently infected, 47% in urban areas and 53% in rural areas. While the national survey in 2004 found that the peak ages for prevalence were 30–34 for both men and women, high levels of prevalence persist up to ages 70–74. When adult infections over the age of 49 are included the total number of adults infected with HIV is estimated to be about 310,000 (299,000–325,000).

### Adult HIV Incidence

The prevalence trend combined with the pattern of progression from new infection to AIDS death indicates that the annual number of new adult infections peaked at around 33,000 in 1995 and declined to about 20,000 by 2002 and is currently around 18,000 (12,000–26,000).

Incidence among those 15–49 is declining, from 3.5% in 2000 to 2.4% in 2007. While the overall population growth rate in Botswana has declined to about 1.7% today, the annual growth rate among all adults is still 2.4%. Therefore, the overall absolute number of new infections each year is approximately constant.

### Adult AIDS deaths and the effects of ART

**Figure 2:**
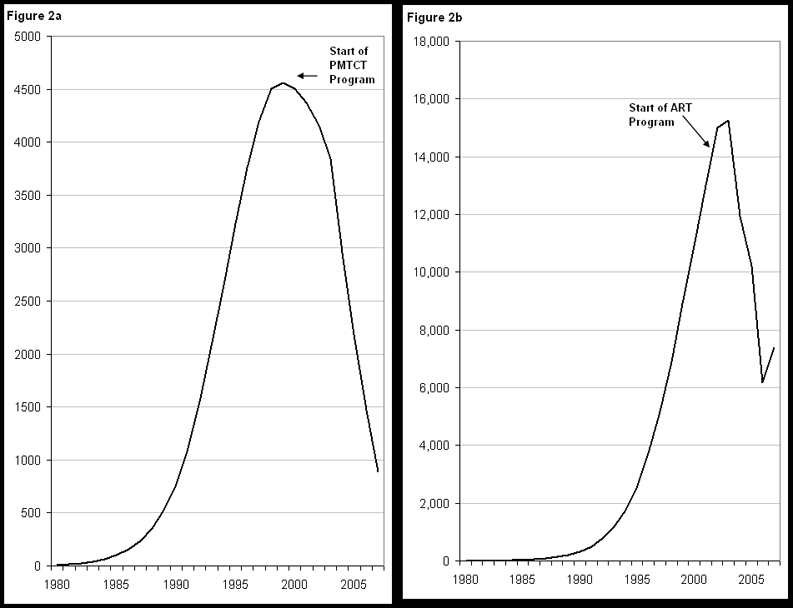
Annual number of new child infections (Figure 2a) and adult deaths (Figure 2b), 1980–2007.

The annual number of adult AIDS deaths rose steadily during the 1980s and 1990s to a peak of nearly 16,000 in 2003. Without any ART program adult AIDS deaths would have continued increasing to nearly 23,000 in 2007. However, Botswana has implemented a vigorous treatment program that has expanded the number of adults receiving ART from 932 in 2000 to 85,497 by the end of 2007, about 83% of need [11]. As a result the estimated number of adult AIDS deaths has dropped to 7400 (5000–11,000) in 2007 (Figure 2b). The provision of ART has averted an estimated 53,000 deaths from 2000 to 2007. If these deaths had not been averted by ART then AIDS deaths would have exceeded new infections and adult HIV prevalence would have been much lower. We estimate that prevalence would have declined to 22.7% in 2007, three percentage points lower than the actual estimate.

Those currently receiving ART will continue to need it for many years to come. In addition, about 23,000 adults newly progress to need for ART each year. As a result the need for ART is expected to increase by almost 60% from 120,000 (101,000–136,000) in 2007 to about 190,000 by 2016 if the high levels of coverage are maintained. This has important cost and sustainability implications for the programme.

### Child infections and the effects of PMTCT

The high levels of HIV infection in adults mean that many children are exposed to the risk of acquiring infection from their mothers. The estimated number of new infections among children peaked at around 4600 in 1999. PMTCT services were introduced in 2002 and coverage expanded rapidly. By 2007 91% of HIV-positive pregnant women received antiretrovirals to reduce the risk of mother-to-child transmission [12]. More than half of these women receive a combination of single dose Nevirapine and AZT. The program carefully tracks women attending antenatal care and women giving birth recording HIV status and for those HIV+ women it reports the number of women evaluated for ART, eligible for ART, already on ART, and use of AZT, Nevirapine and co-trimoxazole. Since early 2007 the program has tracked outcomes for children born to HIV-positive mothers by using dried blood spots and PCR tests on all children born to HIV+ mothers. The results to date indicate that the program has reduced the average mother-to-child transmission rate to just 3.7% by 2007 [12]. Using this figure we estimate that the annual number of new child infections is now about 890 (810–980) (Figure 2a). The expansion of the PMTCT program has averted 10,000 child infections from 2002 to 2007.

HIV-positive children have benefited from an expansion of treatment programs. By the end of 2007 7,400 children were receiving ART, nearly all those estimated to be in need, and almost 10,000 were receiving co-trimoxazole. As a result child AIDS deaths have been reduced from about 3,000 in 2001 to 790 (600–1020) in 2007. Nearly 11,000 child deaths have been averted by the combined effects of PMTCT, child treatment and adult ART.

### Orphans

As a result of adult AIDS deaths a significant proportion of children have lost one or both parents. We estimate that there were 130,000 (110,000–150,000) orphans in 2007, about 16% of all children under the age of 18. Three-quarters of these orphans are due to AIDS. One-third of households caring for orphans are receiving external support [12]. The success of the adult ART program has reduced the number of new orphans each year by 40%, from 20,000 in 2002 to 12,000 in 2007.

## Discussion

Botswana has an HIV surveillance system and a national HIV survey that provide the basis for estimates of the extent of the HIV epidemic and its dynamics. These data indicate that prevalence is currently declining slightly in urban areas and stable in rural areas. The estimates produced from these data indicate that the number of new infections rose rapidly during the early 1990s, peaking in the mid-1990s before falling to a stable level of about 18,000 new infections per year today. The number of AIDS deaths started to grow rapidly about 10 years later than the rise in new infections, peaking just before the expansion of ART.

The successful expansion of the ART program has increased coverage to over 80% of those in need of treatment. This expansion has had significant benefits, reducing the annual number of AIDS deaths by half and, as a consequence, also cutting in half the number of new orphans each year. The high coverage of adult ART has also contributed to a reduction in mother-to-child transmission of HIV.

Due to the high number of new infections in the past 24,000 adults progress to eligibility for ART each year. As a result, the need for adult ART will increase by nearly 60% by 2016. This presents a major challenge to maintain the current levels of high coverage.

The PMTCT program represents a major success with over 90% of HIV-positive women receiving antiretrovirals to prevent transmission of HIV to their children. The program has averted an estimated 10,000 child infections since its inception. The combined effects of the PMTCT program and the child treatment program have averted an estimated 11,000 child AIDS deaths. With fewer new child infections the need for child treatment is also be reduced.

**Figure 3:**
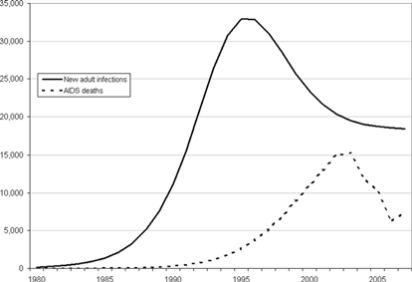
Annual number of new adult HIV infections and AIDS deaths, 1980–2007.

Unfortunately similar progress has not been made in reducing the number of new adult infections (Figure 3). Some prevention programs have been expanded to scale. Condom use in Botswana is among the highest anywhere in the world. High coverage has been achieved for voluntary counseling and testing and AIDS education in the schools. But these programs have not been enough to make a significant difference. The proportion of adults with more than one sexual partner remains very high. A high level of partner concurrency contributes to rapid reproduction of new infections. New approaches are urgently needed. At the current rate of new infections prevalence will remain at very high levels and the burden to expand treatment programs in the future will continue to grow.
